# Book Reviews

**Published:** 1895-11

**Authors:** 


### *BOOK REVIEWS.*

---

THE URINE IN HEALTH AND DISEASE, AND URINARY ANALYSIS, PHYSIOLOGICALLY AND PATHOLOGICALLY CONSIDERED. By D. Campbell Black, M.D., L.R.C.S., Edin., Professor of Physiology in Anderson's College Medical School; Physician to the Glasgow Public Dispensary (department for kidney and urinary diseases). Philadelphia: Lea Brothers & Co.

The great number of books on urinary analysis which have appeared in the last few years is evidence of the constantly growing interest which is felt by the general practitioner in this very important subject. The opening chapter in the book before us is devoted to a consideration of the anatomy and physiology of the kidney, in which is included several theories which have been advanced to explain urinary excretion: while this is well written, it is questionable if it should properly find a place in a work of this kind.

Next follows several chapters in which the normal constituents of the urine are very fully considered; the text is often elucidated by excellent cuts. The remainder of the book is devoted to the study of urinary abnormalities. Attention has been given especially to the best and most modern methods of recognizing abnormal substances which appear in the urine in disease, and the conclusions as to the relative efficacy of these methods seems on the whole just and warranted by the facts. This part of the work is, however, not wholly unexceptionable. We see, with much surprise, that the author considers both the heat and nitric acid tests for albumen more delicate than either the picric acid or the acetic acid and ferrocyanide of potassium tests! Again, in speaking of the phenyl-hydrazin test for sugar, we note that he fails to call attention to the fact that recent investigations have shown that glycuronic acid may cause a precipitate which closely resembles that produced by sugar. The closing chapters contain instructions for analysis of calculi, and a list of the drugs which may be found in the urine after their ingestion. The publishers' work is most excellent. On the whole, this book may be considered the equal of any one of its class. H. F. H.

---

A TEXT-BOOK OF PRACTICAL MEDICINE. Designed for the use o Students and Practitioners of Medicine. By Alfred L. Loomis, M.D., LL.D., Professor of Pathology and Practical Medicine in the Medical Department of the University of the City of New York; Visiting Physician to Bellevue Hospital, etc. Revised and enlarged, with two hundred and seven illustrations and one chromo-lithographic plate. Eleventh edition. Price, cloth, $6.00; leather, $7.00. William Wood & Co., 45-47 East Tenth street, New York.

The fact that Professor Loomis's work has passed through eleven editions in as many years, is sufficient to attest the position which it occupies with students and practitioners. The distinguished author was actively engaged in this revision at the time of his last illness; in fact, the greater part of his task had been finished. This volume contains the results of his later studies in the diseases to which he largely devoted his life-work, namely, the disease of the heart and lungs. Some chapters have been entirely rewritten. At the author's request, Dr. C. G. Coakley and Dr. E. D. Fisher, of the University of the City of New York, have revised and rewritten the sections on the nose and throat, and nervous diseases, respectively. Among the new diseases, we find descriptions of brachycardia, tachycardia, muscular dystrophy, syringo-myelia and acromegaly.

This revision will place in the hands of both practitioner and student a standard work on practice which leaves nothing to be desired. It goes without saying, that it will well sustain the popularity so well earned by former editions.

---

A TREATISE ON NERVOUS AND MENTAL DISEASES. By Landon Carter Gray, M.D., Professor of Diseases of the Mind and Nervous System in the New York Polyclinic. New (2d) edition. In one very handsome octavo volume of 728 pages, with 172 engravings and 3 colored plates. Cloth, $4.75; leather, $5.75. Lea Brothers & Co., Publishers, Philadelphia.

The kind reception which the first edition of this work received three years ago has stimulated the author to make the present issue still more acceptable. Every portion has been carefully revised, and new chapters added on Dementia, Confusional Insanity, Delirium, and Massage. The anatomical introduction has been entirely rewritten, in accordance with advances in our knowledge of the anatomy and physiology of the cerebro-spinal system. A useful feature of this work is the inclusion of the medico-legal aspects of nervous and mental diseases, such as railway injuries and other traumatic diseases. One of the most satisfactory chapters is on the neuroses, including epilepsy, migraine, hysteria, chorea, etc. Throughout his book the author has included only such matter as has stood the test of time and experience, and hence its teachings may be depended upon. It is a book for the general practitioner rather than for the nervous specialist who desires exhaustive discussions. We cheerfully recommend it as a sound and practical treatise on this branch of medicine.

---

A TEXT-BOOK ON NERVOUS DISEASES. By American Authors. Edited by F. X. Dercum, A.M., M.D., Ph.D., Philadelphia. 431 engravings and 7 colored plates. Published by Lea Bros. & Co., Philadelphia.

A just criticism of any book must presuppose a careful study of same. Having studied this work, as carefully as within my power, I can justly give my views as to its merits or demerits.

The editor opens with a very graceful and reassuring preface.

The publishers have done well their part.

That this work should interest is not surprising, after reading the long list of contributors. That it should be instructive and up to date is assured, knowing the ability of these gentlemen.

Dr. Dercum exhibited rare judgment in selecting his collaborators, and they, without exception, have produced a great work. It were vain to endeavor to whom to give the wreath, and vainer to attempt to analyze all the material herein treated.

Certainly great time and research have been expended in the production of this collaboration.

Its study is delightful, not only from the interest excited by each especial contributor, but also from the contrast of styles and expression.

The general result after completing the study of this volume is one of satisfaction, and I am sure it will meet with a favorable verdict from the profession. H. H.

---

CLINICAL LECTURES ON DISEASES OF THE NERVOUS SYSTEM. Delivered at the Hospital for the Paralyzed and Epileptic, London, by W. R. Gowers, M.D., F.R.S., Physician to the Hospital, etc., etc. Published by P. Blakiston, Son & Co., Philadelphia.

Than the clinical lecture there is no more instructive method of education. Than Mr. Gowers there is no more practical writer ; hence it must follow that those whose opportunity it is to read this little volume will do so with great profit. There is not a lecture wise advice.

Especially was I impressed with lectures II., VI., IX., X., XIII. and XVI., though I will not allow my especial impressions to bias me in their favor, when the whole twenty lectures are so ably, naturally and clearly given.

Having had the privilege of being present at many of Dr. Gowers' clinics, I am possibly over zealous in proclaiming the intrinsic merit of the lectures; but as my memories carry me back to the afternoon lecture hour in old Queen's Square, London, and knowing the wonderful field of observation, the great capacity for work and earnest love for his profession with which Dr. Gowers is blessed, I cannot recommend any work from his pen too heartily.

H. H.

MODERN MATERIA MEDICA, with Therapeutic Notes. For the Use of Practitioners and Students of Medicine. By Dr. Otto Roth. Seventh edition. Revised by Dr. Gregor Schmitt, Würzburg. One volume of 467 pages, octavo, muslin binding. Price, $2.00. William Wood & Co., Publishers, New York.

This book formerly appeared as a translation for Wood's Medical and Surgical Monographs. It has proven so popular at home and abroad that a new translation of the seventh German edition is offered as an independent volume. The revision and translation brings the subject of Materia well up to the present time, including within this book that is not teeming with practical suggestions and a conservative discussion of late additions to our pharmacopœia. An attempt has been made to be as concise and practical as possible. Indeed, we think this attempt has been carried, perhaps, too far. Particularly is this true in the descriptions of drugs, and of the plants or other sources from which they come. The book contains a large number of prescriptions. One hundred and thirty-six pages are devoted to "Therapeutic Notes," in which the management of different diseases is concisely outlined.

---

A MANUAL OF SURGICAL ASEPSIS. By Carl Beck, M.D., Visiting Surgeon to St. Mark's Hospital, New York. Well illustrated. Price, $1.25. W. B. Saunders, Publisher, 925 Walnut street, Philadelphia.

The leading idea of the author in writing this book has been to furnish the reader with a compact and practical manual of surgical asepsis, giving a brief statement of the theory upon which modern aseptic methods are based, and a detailed description of the means of securing asepsis. The first chapter discusses the influence of microbes in producing surgical diseases, and in wound infection. The author next describes the means of disinfection, as applied to the field of operation, the operator, instruments and dressings, the sterilization of ligatures, sponges, etc. Other chapters describe the aseptic operating-room, infected wounds, aseptic wounds, aseptic wound treatment, removal of dressings, technique of an aseptic operation, etc. One of the best chapters tells how to obtain asepsis in private practice. The book contains numerous illustrations, and twelve full-page plates. Surgeons will find this work very helpful.

